# Social influence and political mobilization: Further evidence from a randomized experiment in the 2012 U.S. presidential election

**DOI:** 10.1371/journal.pone.0173851

**Published:** 2017-04-26

**Authors:** Jason J. Jones, Robert M. Bond, Eytan Bakshy, Dean Eckles, James H. Fowler

**Affiliations:** 1 Department of Sociology, Stony Brook University, Stony Brook, New York, United States of America; 2 Institute for Advanced Computational Science, Stony Brook University, Stony Brook, New York, United States of America; 3 School of Communication, The Ohio State University, Columbus, Ohio, United States of America; 4 Facebook, Menlo Park, California, United States of America; 5 Department of Medicine, University of California, San Diego, La Jolla, California, United States of America; 6 Department of Political Science, University of California, San Diego, La Jolla, California, United States of America; University of Vermont, UNITED STATES

## Abstract

A large-scale experiment during the 2010 U.S. Congressional Election demonstrated a positive effect of an online get-out-the-vote message on real world voting behavior. Here, we report results from a replication of the experiment conducted during the U.S. Presidential Election in 2012. In spite of the fact that get-out-the-vote messages typically yield smaller effects during high-stakes elections due to saturation of mobilization efforts from many sources, a significant increase in voting was again observed. Voting also increased significantly among the close friends of those who received the message to go to the polls, and the total effect on the friends was likely larger than the direct effect, suggesting that understanding social influence effects is potentially even more important than understanding the direct effects of messaging. These results replicate earlier work and they add to growing evidence that online social networks can be instrumental for spreading offline behaviors.

## Introduction

A number of observational network studies suggest that offline behaviors spread in networks via social influence [1–8]. However, causal inference in observational data can be difficult because social influence, friendship selection, and contextual effects all generate similar patterns in network data [9–10]. For this reason, scholars have complemented these observational studies with experimental studies that use randomization to ensure that what is being measured is, indeed, social influence [11–15].

In particular, we previously conducted an experiment to measure social influence in the 2010 U.S. Congressional Election [11]. That study randomized get-out-the-vote (GOTV) messages to 61 million Facebook users, 6 million of whom were matched to publicly available voter registration records. The results showed that the message directly influenced about 60,000 additional people to vote in 2010. The study also compared the voting behavior of the friends of those who received the message and the friends of those who did not and found that the message indirectly influenced an additional 280,000 people to vote. This social influence effect was limited to “close friends” who interact frequently on Facebook and who likely also had strong, real-world, face-to-face relationships [16]. The results provided evidence that online social networks could spur social influence offline and did so primarily by activating offline social relationships.

An open question that remained after that study was whether such messages would result in similar effects in a U.S. Presidential Election. It is well known that get-out-the-vote messages are less effective during high-stakes elections [17]. Since more people participate in Presidential Elections, there are fewer people to mobilize, and the few who do not participate are bombarded by increased outreach from candidates, parties, and interest groups. According to the Federal Election Commission, total spending on campaigns was about $4 billion in the 2010 U.S. Congressional Election but that increased to about $7 billion in the 2012 U.S. Presidential Election [18]. Voters' behavior and attitudes suggest they consider it more important to be informed and to vote in Presidential election years than in midterm elections. In June of 2010 Pew reported that only 49% of those surveyed were following news about the election very or fairly closely [19], while in June of 2012 72% reported following election news very or fairly closely [20]. (June was the latest month for which we could find comparable figures in both 2010 and 2012 concerning the extent to which an individual was following campaign news.) Gerber et al. [21] report that 78.5% of survey respondents felt that the outcome of a House of Representatives general election (midterm election) would have a big effect on their life, while 83.5% felt that a Presidential general election would have a big effect on their life. Similarly, the authors report that 65.5% would feel bad if they were unable to vote in a House or Representatives general election, while 73% report they would feel bad if they were unable to vote in a Presidential general election. These data underscore how voters view Presidential elections to be of greater importance than midterm elections. Because of this, we were interested in replicating our study in a general election in order to better understand how a changed electoral landscape might impact the effectiveness of get-out-the-vote messages.

It was also unknown whether get-out-the-vote messages on Facebook would continue to have similar effects from one election to the next, as both the Facebook platform, and how voters and campaigns use the service change over time. A recent analysis of Google Flu Trends, an algorithm that predicts real world flu cases based on the frequency of flu-related searches, suggested that the algorithm stopped working in part because it did not adapt to important changes in online search and social ecologies that were constantly evolving [22]. For this reason, it is especially important to replicate scientific studies based on “big data” from online platforms, and in particular, it is important to understand the specific mechanisms of these platforms that drive the behavior. Here, we report the results of a follow up get-out-the-vote experiment we conducted on the day of the 2012 U.S. Presidential Election.

## Method

The research design was reviewed and approved by the University of California, San Diego Institutional Review Board.

**Fig 1:**
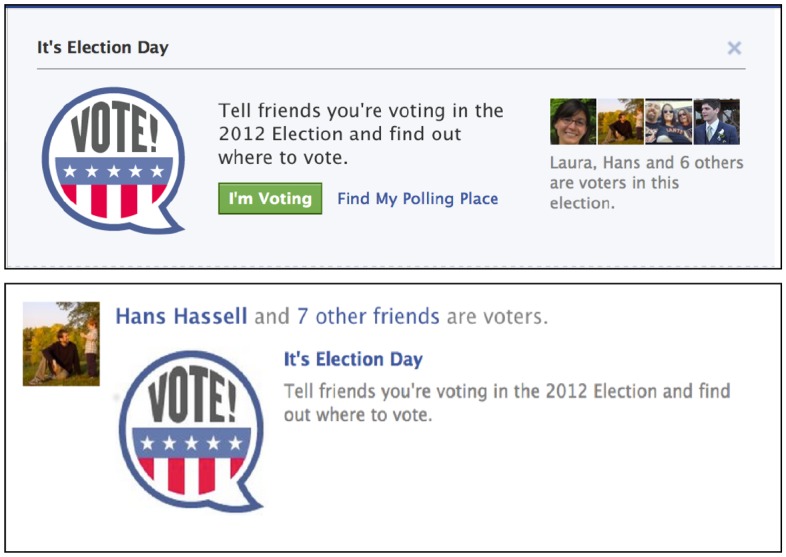
Example messages shown to adult Facebook users in the United States on election day 2012. The top message was shown to users in the “banner” condition at the top of their News Feed. The bottom message was shown to users in the “feed” condition within their news feed if at least one of their friends in the “banner” condition had clicked on the “I’m Voting/I’m a Voter” button.

The experiment employed a 2 x 2 between-subjects design with two different treatment factors designed to measure the effect of different parts of the messaging system employed. The “banner” treatment was intended to measure the impact on voter behavior of seeing a message delivered directly from Facebook. People in this condition saw a banner (Fig 1) above their Facebook News Feed (where users see a list of their friends’ posts) while the control group did not. The banner contained a reminder that it was Election Day, a link to look up local polling places, a button users could press to tell friends they had voted, and a list of up to four of the user’s friends who had already reported voting.

In the 2010 experiment, everyone who saw the banner message was also eligible to see messages within their News Feeds about friends who had used the banner to report they had voted. It was therefore not possible to discern how much of the total effect of the message was due to seeing the banner and how much was due to seeing stories about friends who had voted in the news feed. In the follow up experiment we created a “feed” treatment that was separate from the “banner” treatment in order to measure the impact on voter behavior of seeing a message about friends’ behavior in a feed of other stories independent of the banner. People in the “feed” condition saw messages in their News Feed if their friends interacted with the banner (Fig 1), while the control group did not. These stories appeared in the feed along with other stories (e.g. friends’ status messages, photos, and shared links) and conveyed the information that one or more specific friend had voted.

To ensure the Election Day experience was consistent for most users, the assignment to cells in the design was uneven in favor of the banner and feed conditions (Table 1). Treatments were randomly assigned using PlanOut [23] (see S1 File). In total, 254,223,053 Facebook users were eligible for the experiment on Election Day 2012. Users were eligible if they were 18 years old or older and listed a U.S. state as their state of residence. From 13 states (Arkansas, California, Connecticut, Florida, Kansas, Kentucky, Missouri, Nevada, New Jersey, New York, Oklahoma, Pennsylvania, and Rhode Island) we collected 69,305,265 public records of registered voters and whether they voted in the 2012 election. These 13 states are those that make publicly available voting records that include the variables necessary to match subject records. Specifically, the necessary variables were first name, last name, state of residence and full birthdate. The list of states is the same as was used in the 2010 experiment, and in total these states account for about 40% of all registered voters in the U.S. After we removed duplicates in both the Facebook data and the public voter data (accounting for <0.3% of each sample), 15,060,897 records matched exactly in their first name, last name, state of residence, and full birthdate. Of these, 10,155,987 records indicated the user had logged in on Election Day. Additional users with the Facebook mobile app were eligible to receive a push notification about Election Day, which they were assigned to receive if they were randomly assigned to the banner treatment. Due to technical limitations, we did not observe the eligibility to receive this push notification, and so the randomization to the banner treatment is not valid once conditioning on logging in on Election Day; this is because receiving the notification caused some users to log into Facebook who would not otherwise have done so. This is manifest in a statistically significant difference, among users who logged in, in age between those in the Banner condition and those not. Thus, we focus on the intent-to-treat (ITT) effect on the larger population of all individuals who matched with the public voter data (15,060,897). Compared with the analysis of the 2010 Election, which estimates the effect on only those who log in, our estimates are expected to be smaller and more noisy (because we consider the effect on those who may have never logged on to Facebook or have seen a push notification on election day). However, due to the risk of confounding, we believe it is appropriate to consider this broader population and provide a conservative estimate of the effects on a population for whom we are confident that randomization was achieved. We also consider rescalings of these estimates below to make them more comparable with 2010.

**Table 1:** Percentage of participants assigned to each condition in the 2 x 2 design.

	*Banner Condition*	*Control*
*Feed Condition*	96%	1%
*Control*	2%	1%

## Results

**Fig 2:**
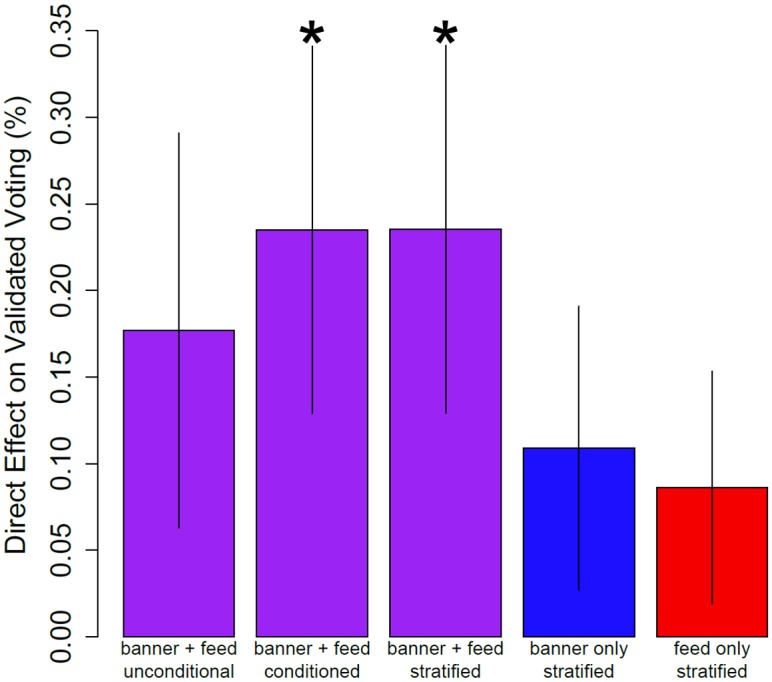
Direct effect of banner and feed conditions on validated voting in 2012. From left to right estimates are based on a regression with heteroskedasticity-robust sandwich standard errors of validated vote on 1) an indicator variable for those in both the banner and feed condition, 2) an indicator variable for being in the banner and feed conditions and a control variable indicating whether the subject voted in 2010, 3) an indicator variable for being in the banner and feed conditions, stratified by voter behavior in 2010, 4 & 5) two indicator variables, one for being in the banner condition and one for being in the feed condition, stratified by voter behavior in 2010. Regressions in 1)– 3) exclude individuals who received only one of the two treatments so the comparison is both vs. neither. These results suggest the banner and feed condition combined to yield a 0.24% increase in voter turnout, and that it likely depended on both mechanisms to generate this increase. Asterisks indicate *p*<0.05.

Fig 2 summarizes the main results. The 2010 experiment did not have separate treatments for banner and feed, so for the 2012 experiment we first measure the direct effect of being in both banner and feed treatments (N = 14,458,236) compared to being in neither (N = 150,139). The untreated group had a validated turnout rate of 73.91% compared to 74.08% for the treated group. A simple regression of turnout on an indicator variable for receiving both treatments increased the likelihood of voting by +0.17% (the 95% confidence interval [CI] for the coefficient in the regression is –0.05% to +0.4%) but this estimate is too noisy to yield confidence that it is different from chance. To increase precision, we include in the regression past voting behavior in 2010 (encoded as two dummy variables, voted and abstained, with unknown status as the baseline category). This yields an estimated treatment effect of +0.24% (95% CI +0.03% to +0.44%) that is unlikely to be due to chance (*p* = 0.0266). We also estimated a post-stratified model that included separate average treatment effects for those who voted, abstained, or had unknown behavior in the 2010 election and combined these, weighting on the number of individuals in each stratum. This procedure provides additional robustness due to the different sized experimental groups and potential for subgroup heterogeneity [24]. These estimates yielded nearly identical average treatment effect of +0.24% (95% CI +0.03% to +0.44%, p = 0.0266). For better comparability with the 2010 election, we also rescaled the post-stratified estimator of the ITT by the proportion of users in the treatment who logged in (i.e., the Wald IV estimator); this yields an estimated local average treatment effect of 0.35%.

**Fig 3:**
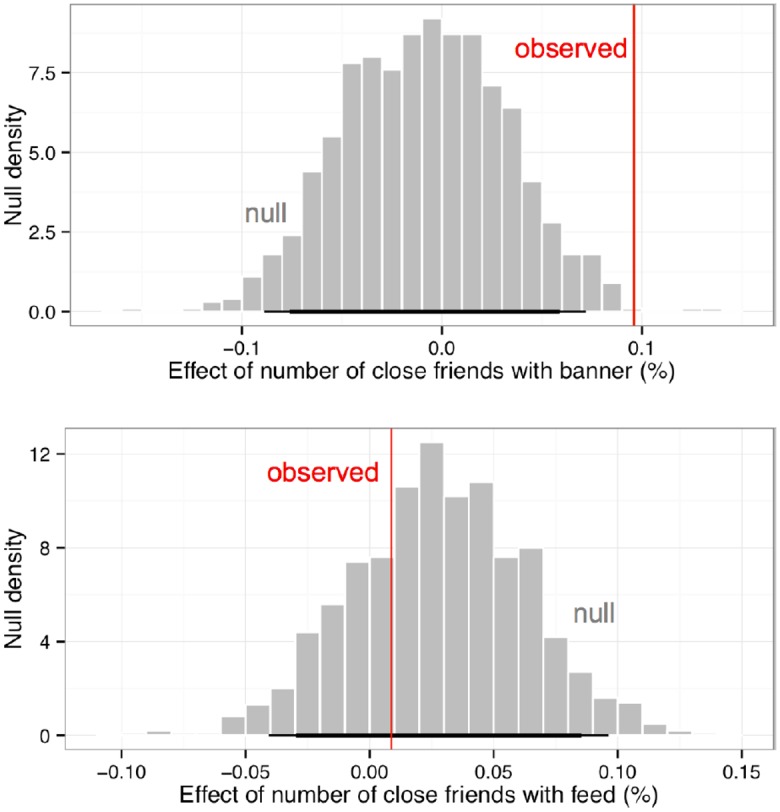
Observed increase in the probability of voting caused by each additional close friend in the banner (top) and feed (bottom) treatments are shown in red. A null distribution of possible outcomes when the network structure is fixed but the treatments are randomly permuted is shown in gray. The results suggest that the banner treatment was effective in spreading behavior to friends (a 0.1% increase in voting likelihood for each close friend treated), but the feed treatment was not. Stratified statistical analyses also replicate these results.

Fig 3 also shows our attempt to discern whether the joint treatment effect was driven by seeing the banner or seeing stories in News Feed about other people who had clicked on the “I voted” button. We included the whole sample (N = 15,060,695) in a regression of individual turnout on separate indicator variables for receiving the banner treatment, for receiving the feed treatment, for voting in 2010, and for abstaining in 2010 (with unknown status in 2010 as the baseline). These results suggest that the treatment effect for the banner was +0.12% (95% CI –0.08% to +0.32%) and the treatment effect for the feed was +0.10% (95% CI –0.06% to +0.26%), though neither of these were significantly different from chance. A stratified model yielded similar results, suggesting that neither the banner nor the feed was solely responsible for the significant joint treatment effect we observed.

Given a direct effect of the banner and feed treatments on voting, to what extent did they indirectly influence the *friends* of those who were treated? Consistent with analysis of the 2010 experiment, we focused on friends who communicated the most with one another, since these are likely to be relationships that exist offline between “close friends” [16]. For each person in the analysis, we counted the number of actions (e.g., comments, tags; see S1 File for the list of actions) that person directed towards each of their friends and computed the fraction of their actions directed to each. For those friends with at least one action, we computed the percentile of the fraction of actions directed to that friend. Friends in the 90^th^ percentile and above were counted as “close friends.” This procedure yielded an average of about 5 “close friends” per person.

We then regressed individual turnout on the number of close friends who received the banner treatment, the number who received the feed treatment, and whether the individual themself received the banner or feed treatments, stratifying on the total number of close friends and voting behavior in 2010. Fig 3 shows that each additional close friend who received the banner treatment increased the likelihood a person voted by about +0.10%. To discern whether this observed spillover effect was due to chance, we simulated the null distribution of estimated spillover effects we might observe by keeping the friendship network intact and permuting who was treated and who was not across all individuals in the experiment [25–26] (see S1 File). The gray histogram shows the effect sizes we estimate using the same regression framework noted above for each of 1,000 permutations. 99% of these null observations fall between –0.09% and 0.07%, and since the observed value falls outside the distribution, it is unlikely that the observed treatment effect is due to chance.

We repeated these procedures to estimate the effect of the feed treatment on friends. However, the estimated effect size was near zero (+0.01%) and fell in the middle of the null distribution (95% CI –0.04% to +0.10%), suggesting that the feed treatment did not spillover to close friends on Facebook. These results imply that placement of the message in the banner instead of the feed was more effective at generating indirect effects. However, it is important to remember that there were other differences in the messaging due to the context. For example, messages in the feed did not contain a “call-to-action” button to self-report voting or a link to click on to find one’s polling place. To learn more about the underlying causes of the difference between banner and feed, future experiments will need to randomize these features to see which might be driving the difference.

Only the banner treatment generated a “multiplier” effect as it spread from one person to another. We can estimate this multiplier effect by calculating the ratio of the expected friends motivated (this is the average number of friends times the per friend treatment effect) to the direct treatment effect. The results suggest that for every one person directly motivated to vote, an additional 5 x 0.10% / 0.24% ≈ 2 friends were indirectly motivated to vote.

Using the same methodology that we used for the 2010 experiment [11], we estimate the total direct effect of the experiment on turnout as the average treatment effect (0.0024) times the number who were treated and matchable to the voter record (14.5 million) divided by the fraction of the voter record we obtained for matching (0.40). We also estimate the total indirect effect on turnout as the average per-close-friend treatment effect (0.001) times the average number of close friends (5) times the number who were treated and matchable to the voter record (14.5 million) divided by the fraction of the voter record obtained for matching (0.40). Note that this estimate assumes that the treatment effect on voters with unmatchable records (which far exceeded those with matchable records) was 0.

The results suggest that the experiment directly increased turnout by about 90,000 people. Despite the apparent smaller effect sizes in 2012, the total number of people directly mobilized was actually higher in 2012 than it was in 2010 (90,000 vs. 60,000) because the increase in the Facebook population outweighed the (statistically insignificant) decrease in the estimated average effect of the messages. The treatment effects also spread through the network as in 2010, causing an additional 180,000 close friends of the treated to vote as well, for a total increase of 270,000 people voting in the 2012 U.S. Presidential Election.

Replication data is available at the following doi: 10.7910/DVN/J0VEYF

## Discussion

Although get-out-the-vote messages are typically less effective in high-stakes Presidential Elections than they are in Congressional Elections [17], we demonstrate in this article that a single message on Facebook nonetheless motivated a significant number of people and their friends to go to the polls. The per-person direct effect of those who (potentially endogenously) logged into Facebook (0.24%) we estimate for 2012 is similar to the effect of the intervention on those who logged into Facebook on election day of 2010 (0.40%). The differences between the 2010 estimates, ITT estimates from 2012 (0.24%), and LATE estimates (0.34%) from 2012 estimates are not statistically significant.

A consistent finding from both the 2010 and 2012 experiments is that indirect effects account for a large majority of the total impact of get out the vote campaigns. We estimate that the effect of the message on friends accounted for twice as many votes as the direct effect on those who saw the message. That is, the indirect effect accounted for about 67% of the total effect in 2012, compared with an estimated 80% of the total effect in 2010. These results suggest that understanding the mechanisms for the spread of behavior could potentially be more valuable than better understanding the direct effect of messages on individual behavior.

Finally, our comparison of the banner and feed treatments suggests direct messaging may be the best way to stimulate spillover effects since those who saw friends’ voting behavior in their News Feeds were no more likely to vote than those who did not. It may well be that such messages are effective, but since they are just one of many potential messages about the election (e.g., status updates or link shares from friends about the election) in viewers’ News Feeds, they are less likely to have a substantive effect. We conclude that, despite two massive presidential campaigns making increased use of online advertising, a simple message about friends continues to have a substantial effect on civic participation.

## Supporting information

S1 File Vote12_SI_v8_clean. (DOCX)
