# Supplementary material for: Social influence and political mobilization: Further evidence from a randomized experiment in the 2012 U.S. presidential election
Source: PLoS One. 2017 Apr 26;12(4):e0173851. doi: 10.1371/journal.pone.0173851 (PMC5405916; doi:10.1371/journal.pone.0173851)
Supplement: S1 File — (DOCX) [file pone.0173851.s001.docx]

**Supporting Information for**

**Social Influence and Political Mobilization: Further Evidence from a Randomized Experiment in the 2012 U.S. Presidential Election**

Jason J. Jones^1,2*^, Robert M. Bond^3,4^, Eytan Bakshy^5^, Dean Eckles^6^, and James H. Fowler ^7,8^

^1^ *Department of Sociology and* ^2^ *Institute for Advanced Computational Science, Stony Brook University, Stony Brook, NY 11794*^3^ *School of Communication and* ^4^ *Department of Sociology, The Ohio State University, Columbus, OH 43210*^5^ *Facebook, Menlo Park, CA 94025, USA*^6^ *MIT Sloan School of Management, Cambridge, MA 02139, USA*^7^ *Department of Medicine and* ^8^ *Department of Political Science, University of California, San Diego, La Jolla, CA 92093, USA*

^*^ To whom correspondence should be addressed, email: [jason.j.jones@stonybrook.edu](mailto:jason.j.jones@stonybrook.edu)

**1. Assignment procedure**

Individuals and friends were randomly assigned to treatments using the following PlanOut (Bakshy, Eckles, Bernstein, 2014) script. Those receiving the banner treatment have a has_megaphone value of 1, and those with has_feed = 1 are eligible to see “is a voter” messages in their News Feeds (which are generated by friends’ clicking of the “I’m voting” / “I’m a voter” button in their News Feeds). Finally, p_show_friend is the proportion of a particular viewer’s friends who are eligible to appear in the banner or News Feeds.

has_megaphone <- bernoulliTrial(p=0.98, unit=userid); # banner
ma <- bernoulliTrial(p=0.50, unit=userid); 
mb <- bernoulliTrial(p=0.9795, unit=userid);
 mega_probs <- [ma, mb];

has_feed <- mega_probs[has_megaphone];

user_voter <- bernoulliTrial(p=0.5, unit=userid);
friends_voter <- bernoulliTrial(p=1.0, unit=userid);
button_types <- ["voting", "voter"];
button_type <- button_types[user_voter];

p_all_cues <- bernoulliTrial(p=0.6, unit=userid);
uniform_p <- randomFloat(min=0.0, max=1.0, unit=userid);
cue_probs <- [uniform_p, 1.0];
 p_show_friend <- cue_probs[p_all_cues];
friends_shown <- bernoulliFilter(
 p=p_show_friend, choices=friends, salt=’show_friend’, unit=userid
);
friend_shown <- bernoulliTrial(
 p=p_show_friend, salt=’show_friend’, unit=[userid, friend]
);

We had some concern that the treatment affected who logged in to Facebook on Election Day. Users with the Facebook app on their smartphone and who were in the Banner and Feed conditions received notifications regarding their friends’ clicking the "I Voted" button. We believe this made these users more likely to visit Facebook, and this population was more likely to be younger. Balance tests revealed a small difference in age between those in the Banner (37.8 years old) and Control (37.9 years old) conditions (p = 0.003).

To ensure a balanced population, we widened the scope of analysis to all users for whom we had matched voting behavior. The balance test for this population (Table S1) detects no differences due to condition.

**2. List of Actions Included When Determining Which Friends are “Close Friends”**

We included the following directed actions when counting the total number of interactions between each person and each of their friends over the six months prior to Election Day:

- Comment
- Friend
- Tag
- Wall post
- Poke
- Mention
- Profile suggestion
- Add to group
- Follow
- Family relationship
- Contact field
- Relationship
- Add as administrator
- Answer question

A sum of the number of actions directed at another user (in a given time period) is a good proxy for tie strength. The actions listed above have been demonstrated to predict who a Facebook user will name as a “close friend” when asked (Jones, Settle, Bond, Fariss, Marlow & Fowler, 2013).

Frequency of contact in general is a good proxy for tie strength. As examples, subjective tie strength has been shown to correlate with the reciprocity of calls made between two mobile phone users (Zhang & Dantu, 2010) and the number of tweets exchanged between Twitter users (Baatarjav, Amin, Dantu & Gupta, 2010). The broader principle has been referred to as media multiplexity – close ties are likely to use a variety of media to communicate and will have frequent contact over multiple channels.

**3. Randomization Inference for Indirect Effects**

To test for indirect effects (i.e., peer effects) while accounting for network dependence, we used a randomization inference method for non-sharp null hypotheses (Aronow, 2012; Athey, Eckles, Imbens, 2015), in which the observed estimates for indirect effects are compared with their distribution under the null; in particular, the null here is that there may be direct effects of the treatment, but there are no indirect effects. These methods require partitioning the experimental units into *focal units*, for which we condition on their treatment assignment and include their outcome data, and *auxiliary units*, for which we permute their treatment assignment. The resulting p-values are valid for any choice of focal units, but some choices result in more powerful tests (Athey, Eckles, Imbens, 2015). We select all users for which the verified voting outcome is available as focal units.

We keep the network, outcome data, and focal units’ treatment assignments fixed. We then draw a new random assignment to the banner and feed treatments for the auxiliary units. We compute the regression coefficients for focal units’ verified voting on their own treatment and the number of their close friends assigned to feed and the number assigned to banner, stratifying on the number of close friends. We repeat this procedure 1,000 times; each is a draw from the distribution of these coefficients under the null hypothesis that there are no indirect effects.

**4. Priming Voter Identity to Increase Turnout**

Past research (Bryan, Walton, Rogers & Dweck, 2011) has suggested that priming identity via the use of nouns ("I’m a voter") may increase the effectiveness of a get-out-the-vote message relative to priming actions activated by verbs ("I voted" or "I’m voting"). The experimental design included a third “identity” treatment that was designed to measure the impact of an identity frame on voting behavior. In this treatment, the text of the button in the banner condition read either “I’m a voter” or “I’m voting.” No statistically significant effects or interactions for the button text were observed for validated vote. We chose to merge these conditions in the presentation in the main text to ease discussion and interpretation.

|  | **Control** | **Treatment** | **Difference** | ***t*** | ***p*** |
| --- | --- | --- | --- | --- | --- |
| **Banner Condition** |  |  |  |  |  |
| *Friend count* | 316.3 | 317.4 | 1.1 | 1.40 | 0.16 |
| *Age* | 35.216 | 35.210 | –0.006 | –0.18 | 0.86 |
| *Female* | 0.6068 | 0.6074 | 0.0006 | 0.54 | 0.59 |
| **Feed Condition** |  |  |  |  |  |
| *Friend count* | 317.3 | 317.4 | 0.1 | 0.08 | 0.93 |
| *Age* | 35.213 | 35.211 | –0.003 | –0.10 | 0.92 |
| *Female* | 0.6065 | 0.6074 | 0.0009 | 1.02 | 0.31 |
| **Noun Condition** |  |  |  |  |  |
| *Friend count* | 317.3 | 317.4 | 0.1 | 0.45 | 0.65 |
| *Age* | 35.219 | 35.202 | –0.017 | –1.92 | 0.06 |
| *Female* | 0.6073 | 0.6074 | 0.0001 | 0.50 | 0.61 |

**Table S1.** Balance tests show that groups assigned to treatment and control have similar number of friends, age, and gender for each condition. *T* statistics and *p* values based on simple difference of means tests.

**References**

Aronow, P., (2012), A general method for detecting interference between units in randomized experiments. *Sociological Methods & Research*, *41*(1): 3-16.

Athey, S., Eckles, D., Imbens, G. W. (2015). Exact p-values for testing non-sharp null hypotheses with applications to networks. Working paper.

Baatarjav EA, Amin A, Dantu R, Gupta NK (2010) Are You My Friend? IEEE CCNC 2010.

Bakshy, E., Eckles, D., & Bernstein, M. (2014). Designing and deploying online field experiments. *Proceedings of WWW 2014*. ACM.

Jones J.J., Settle J.E., Bond R.M., Fariss C.J., Marlow C., Fowler J.H. (2013). Inferring tie strength from online directed behavior. *PLOS One*, *8*(1), e52168.

Zhang H, Dantu R (2010) Predicting Social Ties in Mobile Phone Networks. ISI 2010, May 23–26, 2010, Vancouver, BC, Canada.
